# Predicting gallstones risk with body composition analysis and machine learning: a dual-center cohort study

**DOI:** 10.1080/07853890.2026.2742549

**Published:** 2026-10-07

**Authors:** Hong-Yu Long, Yong-Mei Wu, Xue Li, Bai-Qing Chen

**Affiliations:** a Department of Interventional Medicine, Jinqiu Hospital of Liaoning province, Shenyang, Liaoning Province, China; b Department of Radiology, Panjin Liaohe Oilfield Gem Flower Hospital, Panjin, Liaoning Province, China; c Department of Radiology, The People’s Hospital of China Medical University, Shenyang, Liaoning Province, China; d Department of Radiology, The People’s Hospital of Liaoning Province, Shenyang, Liaoning Province, China

**Keywords:** Gallstone, visceral adipose tissue, deep learning, computed tomography

## Abstract

**Background:**

To compare the predictive performance of computed tomography (CT) body composition indices, anthropometric indices, and laboratory indices for gallstones occurrence, and to construct machine-learning models to improve performance.

**Methods:**

The dual-center retrospective cohort enrolled patients who underwent initial abdominal CT between January 2017 and January 2023, had no gallstones detected, and completed at least 3 years of follow-up. The data analysis was performed in April 2026. They were divided into gallstone group and non‑gallstone group by follow‑up findings. A deep-learning tool, Body and Organ Analysis (BOA), was used to quantify fat, muscle, and bone at the level of the third lumbar vertebra. The area under the receiver operating characteristic curve (AUC) of these indices was compared with that of anthropometric and laboratory indices. Predictive models were developed in the training cohort. Model performance was evaluated using fivefold cross-validation and an independent test cohort.

**Results:**

1,944 patients were evaluated, including 1,437 in the training cohort (Center 1; median age, 63 years [25th–75th percentile, 55–72]; 699 males) and 507 in the test cohort (Center 2; median age, 63 years [55–71]; 266 males). In univariate analysis, neutrophil-to-lymphocyte ratio (NLR) showed the highest AUC (0.627, 95% confidence interval [CI]: 0.586–0.668). The extreme trees (ET) model performed best, with a test-set AUC of 0.772 (95% CI: 0.714–0.822). SHapley Additive exPlanations (SHAP) analysis identified the area ratio of subcutaneous to total fat as the most important feature.

**Conclusions:**

NLR was the best single predictor but had limited standalone utility. Among models integrating the three indicator categories, the ET performed best.

## Introduction

Gallstones are a common biliary tract disease that imposes a substantial health and economic burden on healthcare systems worldwide [1]. The global prevalence of gallstones diagnosed by imaging is approximately 6.7% [2,3]. Approximately 800,000 cholecystectomies are performed annually in the United States, at a cost of nearly 6.5 billion dollars [2]. With the global epidemic of obesity and metabolic diseases, the incidence of gallstones is rising in parallel [1,4]. Gallstones can lead to serious complications such as acute cholecystitis, and pancreatitis. Recent studies have also found that they are associated with an increased risk of cardiovascular disease, diabetes, and gallbladder cancer, and result in higher mortality [3]. Therefore, early identification of high-risk individuals is crucial to reducing the disease burden.

The formation of gallstones is influenced by multiple factors, with obesity being one of the core risk factors [5,6]. Mendelian randomization studies have confirmed an independent causal relationship between obesity and gallstones [4]. Commonly used anthropometric indices such as body mass index (BMI), waist circumference (WC), body roundness index (BRI), and relative fat mass (RFM) are widely employed for risk assessment due to their convenience and low cost. Multiple studies have confirmed that these indices are significantly associated with gallstones [3,7–9]. One prospective cohort study demonstrated that higher BMI, WC, and waist-to-height ratio independently predicted the risk of new-onset gallstones [10].

However, anthropometric indices have inherent limitations: they cannot distinguish between different body components. BMI cannot differentiate fat from muscle, and WC cannot distinguish metabolically active visceral adipose tissue (VAT) from metabolically inert subcutaneous adipose tissue (SAT) [3,11]. Sekine et al. found that VAT accumulation was significantly associated with gallstones, whereas BMI could not independently predict the risk [12]. A Korean study confirmed that an elevated visceral‑to‑subcutaneous fat ratio was independently associated with an increased risk of gallstones [13]. With advances in imaging technology and artificial intelligence, computed tomography (CT) can precisely quantify VAT, SAT, muscle, and bone at the L3 level or even throughout the entire abdomen [12,14]. Current evidence indicates that the L3 level is highly correlated with whole‑body tissue volumes and represents a reliable site for body composition assessment [15].

In addition to obesity-related indices, recent studies have suggested that inflammation and bilirubin metabolism also play important roles in the development of gallstones [16,17]. The neutrophil-to-lymphocyte ratio (NLR), a marker of systemic inflammation, has been shown to be associated with an increased risk of gallstones [18]. Mildly elevated total bilirubin (TBIL) levels have also been reported to independently predict stone formation [19].

Many cross-sectional studies have investigated the associations between various indices and gallstones. A few prospective studies have validated the predictive value of traditional indices for gallstone occurrence. However, existing studies have largely been limited to analyses within single categories of indices, lacking head-to-head comparisons of body composition, anthropometric, and laboratory indices. Moreover, given the limited performance of individual indices, it is necessary to introduce machine learning (ML) methods and construct multi-indicator prediction models to improve predictive performance.

In recent years, ML has emerged as a promising tool in disease risk prediction, with ensemble methods such as random forest (RF), XGBoost, and extreme trees (ET) demonstrating particular utility in clinical tasks by capturing complex interactions among predictors [20–22]. Several studies have applied ML techniques to gallstone detection. For instance, Sarker et al. proposed a RF classifier optimized by Sand Cat Swarm Optimization algorithm for gallstone classification using clinical data [23]. In addition, several studies have developed ML models that incorporate laboratory indices and anthropometric parameters [24,25]. However, most of these studies are cross-sectional in design and focus on gallstone detection rather than prospective risk prediction. Furthermore, they rarely incorporate imaging-derived body composition parameters, and systematic comparisons across different categories of predictors remain limited (Table 1).

**Table 1.** Comparison of previous gallstone prediction studies and the present study.

Study	Design	Diagnostic basis	Predictors	Algorithm	AUC	Main limitations
Sarker et al. [23]	Cross-sectional	Medical records (319 participants; 161 with gallstones)	Demographic variables, routine clinical laboratory	RF-based ML	0.877	Cross-sectional design cannot establish causality; single-center
Deng et al. [24]	Cross-sectional	Self-report (7,868 participants; 830 with gallstones)	Demographic variables, physical examination, laboratory, and dietary nutrient intake indices	LR, GNB, MLP, SVM	0.727	Cross-sectional; self-reported diagnosis with recall bias and misclassification
Zhao et al. [25]	Cross-sectional	Self-report (8,694 participants; 910 with gallstones)	LFWHR, demographic variables, physical examination, medical comorbidities	LASSO, LR	0.767	Cross-sectional; self-reported diagnosis
Zhou et al. [31]	Cohort	Primary Care, Hospital admissions, and Self-reports (459,523 participants; 14,951 with gallstones)	Demographic variables, lifestyle factors, disease information, multiple obesity indices	Cox proportional hazard model, competing risk model	N/A	Association study rather than predictive modeling; no AUC reported; no machine learning or external validation
The present study	Retrospective dual-center cohort	Abdominal CT (1,944 patients; 310 gallstones)	Demographic variables, CT-based body composition, anthropometric, and laboratory indices	Weighted logistic regression, RF, XGBoost, CatBoost, SVM, ET	0.772	Retrospective design; CT may miss isodense or small stones

AUC area under the receiver operating characteristic curve, RF Random Forest, ML machine learning, LR logistic regression, GNB Gaussian Naive Bayes, MLP multi-layer perceptron, SVM support vector machine, LFWHR liver fibrosis and waist circumference to height ratio, CT computed tomography, ET extra trees.

Therefore, this study aims to systematically compare the predictive performance of CT‑based body composition, anthropometric, and laboratory indices for the risk of gallstone occurrence, and further to construct logistic regression and machine learning models to enhance predictive ability. The unique contributions of this study are as follows:

- Head-to-head comparison of CT-based body composition, anthropometric, and laboratory indices for gallstone risk prediction within a unified analytical framework.
- Longitudinal dual-center cohort design with at least 3 years of follow-up, establishing temporal relationships rather than cross-sectional associations.
- Systematic evaluation of five machine learning algorithms alongside conventional logistic regression, with external validation in an independent center.

## Materials and methods

This section describes the study design, participant selection criteria, data collection procedures, CT-based body composition quantification methods, feature selection strategies, and the development and evaluation of logistic regression and machine learning models. The overall workflow is illustrated in Figure 1.

**Figure 1.**
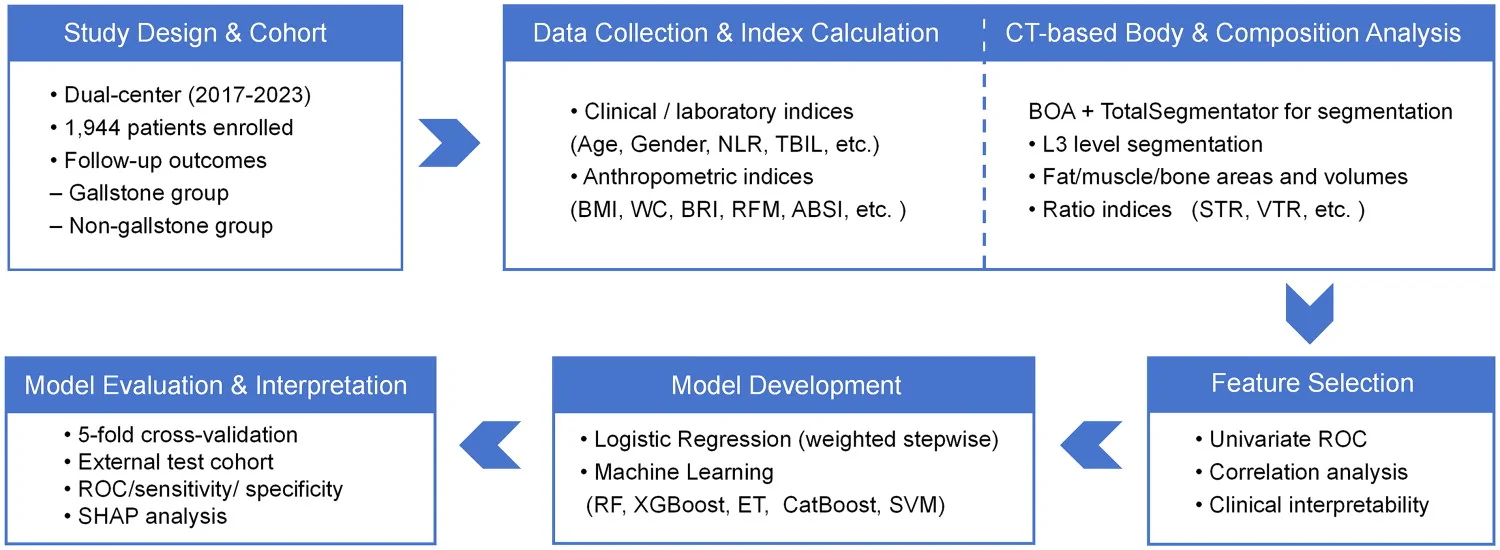
Overview of the study workflow. NLR neutrophil-to-lymphocyte ratio, TBIL total bilirubin, BMI body mass index, WC waist circumference, BRI body roundness index, RFM relative fat mass, ABSI A Body Shape Index, STR subcutaneous adipose tissue/total adipose tissue, VTR visceral adipose tissue/total adipose tissue, ROC receiver operating characteristic, SHAP SHapley Additive exPlanations, RF random forest, ET extreme trees, SVM support vector machine.

### Study population

This retrospective dual-center study was approved by the ethics committees of all participating hospitals. Due to its retrospective nature, the requirement for informed consent was waived (approval numbers: 2025K085, LLSC-2026-KY-14). The training dataset came from Center 1, and the test dataset comprised patients from Center 2. This was a retrospective cohort study that enrolled patients who underwent abdominal CT examination at the two centers between January 2017 and January 2023. Detailed CT equipment parameters are provided in Appendix 1.

Inclusion criteria: (a) age > 18 years; (b) no gallstones detected on the initial abdominal CT examination; (c) follow-up requirements meeting one of the following conditions: for patients ultimately assigned to the non-gallstone group, at least 3 years of follow-up after the initial CT, with at least one abdominal CT examination performed each year during this period; for patients ultimately assigned to the gallstone group, detection of gallstones on at least one follow-up abdominal CT examination within 3 years after the initial CT.

Exclusion criteria: (a) previous or during‑follow‑up history of biliary tract surgery; (b) poor quality of abdominal CT images that precluded body composition segmentation; (c) presence of other conditions affecting the accuracy of abdominal body composition analysis.

A total of 1,944 patients were finally enrolled (Figure 2). The CT diagnostic criteria for gallstones were hyperdense spots within the gallbladder, common hepatic duct, or common bile duct, characterized by well-circumscribed, high-attenuation areas that did not show contrast enhancement.

**Figure 2.**
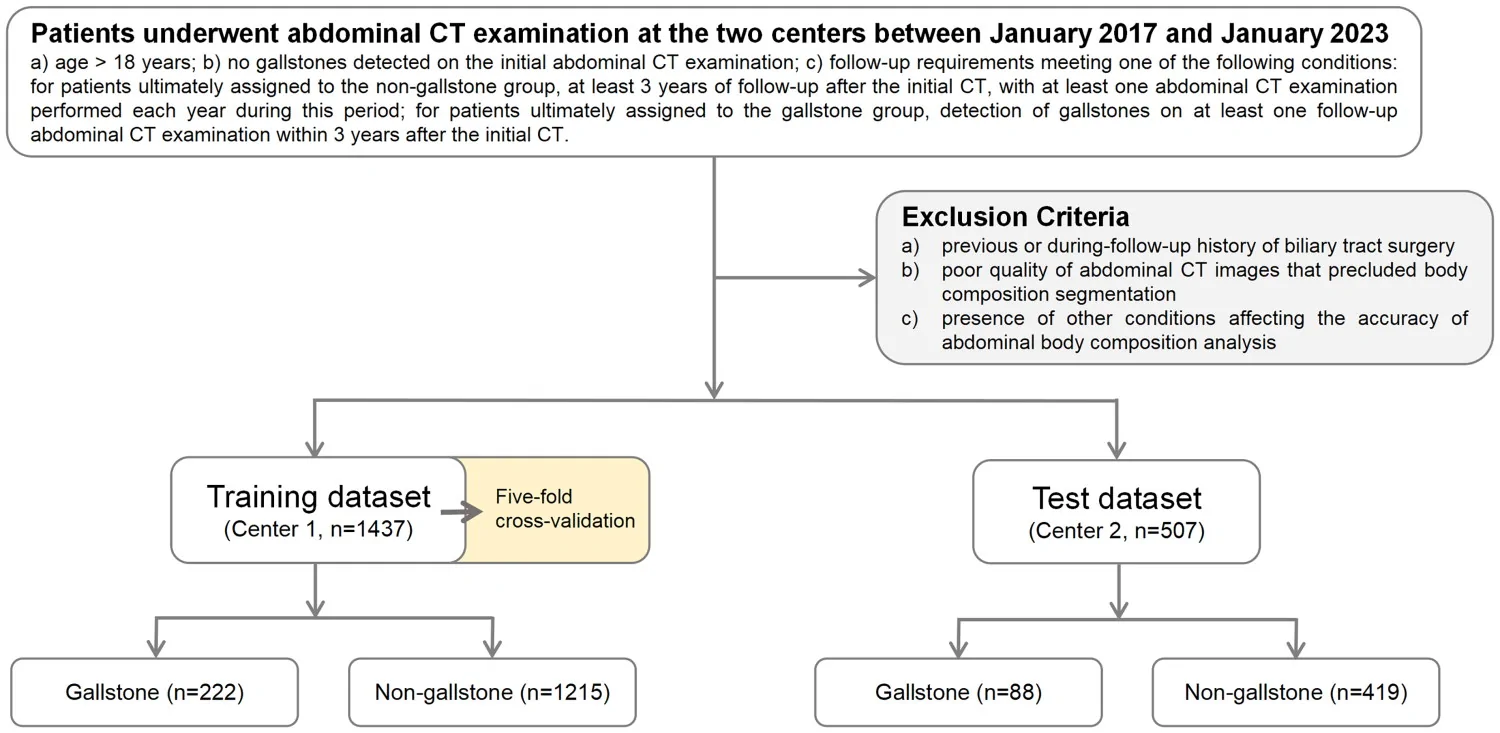
Flowchart study population. CT Computed tomography.

Gallstone group: gallstones were detected on any abdominal CT examination within 3 years after the initial CT. Non‑gallstone group: no gallstones were detected on any abdominal CT examination during a follow‑up period of at least 3 years after the initial CT.

The anatomical distribution of detected stones was further classified according to their location, including isolated gallbladder stones, isolated bile duct stones (common hepatic duct or common bile duct stones), and concurrent gallbladder and bile duct stones. The number of follow-up abdominal CT examinations was recorded for each patient. For patients in the gallstone group, the interval between the initial CT examination and the first detection of gallstones was also calculated.

### Clinical data collection

Medical records closest to the initial abdominal CT (interval ≤30 days) were collected from hospital electronic records. Data included sex, age, height, weight, WC, systolic blood pressure, diastolic blood pressure, fasting blood glucose, fasting triglycerides (TG), fasting high-density lipoprotein cholesterol (HDL-C), total cholesterol (TC), TBIL, indirect bilirubin (IBIL), neutrophil count (NEUT), lymphocyte count (LYMPH), as well as medication use for hypertension and diabetes. The main clinical indications for the initial abdominal CT examinations were retrospectively reviewed based on medical records and categorized into three groups: abdominal pain or abdominal discomfort, tumor surveillance/follow-up, and other indications (e.g. routine health examinations or follow-up for other diseases). WC was measured using bony landmarks on CT images according to the anatomical definition of the International Diabetes Federation (see Appendix 2 for details) [26].

Based on the above data, we calculated BMI, RFM, BRI, A Body Shape Index (ABSI), triglyceride-glucose index (TyG), triglyceride-glucose–body shape index (TyG–ABSI), and NLR. The calculation formulas and the specific diagnostic criteria for hypertension and diabetes are provided in Appendix 3.

### CT-based quantitative analysis and feature extraction of body composition

In this study, a deep learning-based automated tool (BOA), extracted patient body composition parameters [14]. This tool integrates a tissue segmentation model developed on the nnU-Net framework (BCA) and a whole-body anatomical structure segmentation algorithm (TotalSegmentator), enabling automatic identification and quantitative analysis of multiple organ structures [27–29] (Figure 3).

**Figure 3.**
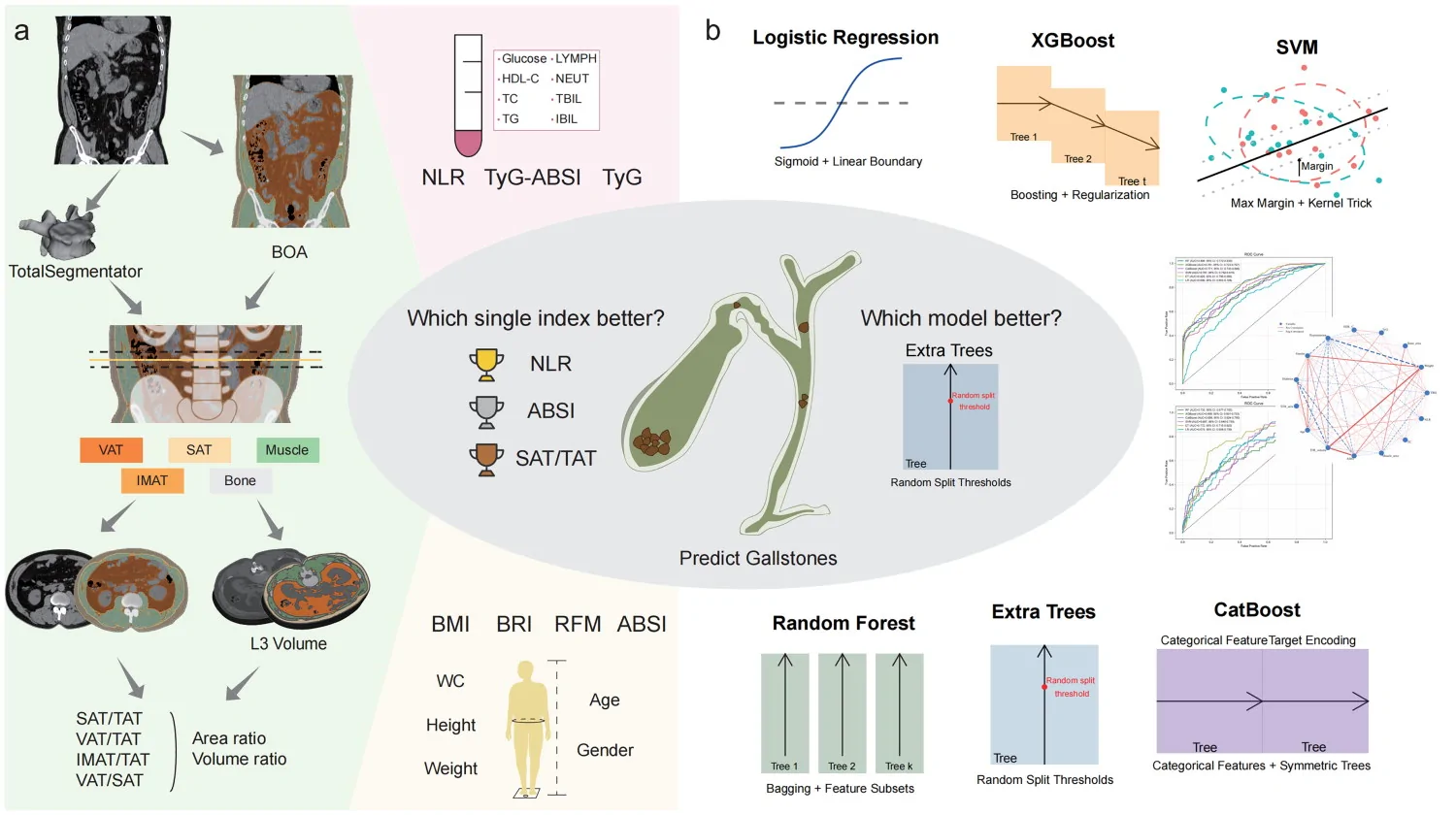
Feature extraction, predictive performance comparison, and predictive model construction. (a) Quantitative parameters of body composition were extracted from abdominal CT images and compared with anthropometric and laboratory indices to evaluate the predictive performance of each single index for gallstone occurrence. (b) Multi-category indices were integrated to construct and evaluate the predictive performance of logistic regression and machine learning models.

The specific workflow for body composition extraction was as follows: First, BOA automatically segmented CT images, obtaining a voxel‑level distribution of adipose tissue (AT) and related body components, including VAT, SAT, intermuscular adipose tissue (IMAT), skeletal muscle, bone. Based on this, the sum of VAT, SAT, and IMAT was defined as total adipose tissue (TAT). Second, TotalSegmentator automatically identified the third lumbar vertebra (L3), determined its upper/lower border slices, and define the mid‑plane as the single‑slice L3 level. Based on this anatomical localization information, a Python script (version 3.7.12) was used to spatially constrain the BOA segmentation results, extracting AT and related body components within the single L3 slice and within the range from the upper to the lower border of the L3 vertebral body. The cross‑sectional areas (VAT_area, SAT_area, IMAT_area, TAT_area, Muscle_area, Bone_area) and total volumes (VAT_volume, SAT_volume, IMAT_volume, TAT_volume, Muscle_volume, Bone_volume) were then calculated. Third, the area and volume ratios of SAT to TAT, VAT to TAT, IMAT to TAT, and VAT to SAT were calculated, including the SAT/TAT area ratio (STR_area), VAT/TAT area ratio (VTR_area), IMAT/TAT area ratio (ITR_area), VAT/SAT area ratio (VSR_area), SAT/TAT volume ratio (STR_volume), VAT/TAT volume ratio (VTR_volume), IMAT/TAT volume ratio (ITR_volume), and VAT/SAT volume ratio (VSR_volume).

### Univariate performance evaluation and feature selection

In the training cohort, the receiver operating characteristic (ROC) curve was used to evaluate the performance of each index in predicting gallstone occurrence. And the indices were ranked in descending order of the area under the receiver operating characteristic curve (AUC). For indices that were highly correlated or belonged to the same category, a preliminary screening was performed based on correlation analysis, predictive performance, and clinical interpretability to reduce feature redundancy and potential multicollinearity. Subsequently, the screened variables were used for feature selection and model building in the multivariate logistic regression and machine learning.

### Development of the multivariate logistic regression model

Due to the imbalance between the gallstone group and the non-gallstone group in the training cohort (222 vs. 1,215), we introduced class weights into the logistic regression (weight for the positive class = number of negative cases/number of positive cases). A fivefold cross-validation was used to evaluate the stability and internal consistency of the weighted model. The AUC, accuracy, sensitivity, specificity, and *F*1 score were calculated for each fold, and the mean ± standard deviation of these metrics served as preliminary performance assessment. Subsequently, the weighted model was refitted on the entire training cohort, and its final discriminative ability, calibration, and clinical utility were evaluated in an independent test cohort.

### Development of machine learning models

We first constructed prediction models in the training cohort. All machine learning models were implemented using the Scikit-learn Pipeline framework, integrating feature preprocessing and classifiers into a unified workflow. Continuous variables were standardized using StandardScaler, and categorical variables were kept in their original encoding. Class weights were dynamically set based on the class distribution in the training cohort to address class imbalance. Five machine learning models were built: RF, XGBoost, CatBoost, Support Vector Machine (SVM), and ET. These models were selected based on their diverse modeling strategies and ability to capture complex relationships between predictors and outcomes (see Appendix 4 for details). For each model, a nested cross-validation framework was used for training and hyperparameter optimization: in the inner loop, a stratified fivefold cross-validation combined with RandomizedSearchCV (50 random searches) was performed, with AUC as the optimization objective to determine the optimal hyperparameter combination for each model. Machine learning modeling and statistical analyses were performed using Python (version 3.10.9). Detailed information regarding the computational environment and software versions is provided in Appendix 5.

Subsequently, the performance of all models was evaluated using stratified fivefold cross‑validation on the training cohort and an independent external test cohort. The model constructed by multivariate logistic regression was included as the baseline model for comparative analysis against the machine learning models. During the evaluation process, metrics including AUC, accuracy, sensitivity, and specificity were calculated, and the discriminative ability of the models was assessed using ROC curves. Confusion matrices and precision–recall (PR) curves were generated to further evaluate model classification performance. Additionally, the bootstrap method (2,000 resampling iterations) was employed to repeatedly sample each performance metric and compute its 95% confidence interval (CI), thereby evaluating the stability and reliability of the model results. Because the training and test cohorts were predefined according to different centers, repeated random train–test splitting was not performed, as it would compromise the independence of the external validation cohort and undermine the validity of the external validation. To assess model stability, repeated fivefold cross-validation was performed on the training cohort using 50 different random seeds (random states 1–50). The performance distribution of the best-performing model was summarized using the mean and standard deviation of AUC.

### Sensitivity analysis

Abdominal ultrasonography findings and surgical records during follow-up were collected for all patients. Sensitivity analysis was then performed on patients whose CT and ultrasound findings were concordant or who had surgically confirmed gallstones. In addition, to assess whether isolated bile duct stones confounded the prediction of gallbladder stones, we performed a sensitivity analysis by excluding these patients, redefining the remaining stone patients as the positive outcome, and retraining and evaluating the model on this refined cohort.

### Statistical analysis

R software (version 3.6.3) was used for statistical analyses. Continuous variables were presented as medians with 25th–75th percentile. Some patients had missing clinical data, with an overall missing rate of <5%. Missing continuous variables were imputed using the median to reduce potential bias caused by missing data. Categorical variables were presented as numbers and percentages (%). Group comparisons were performed using the Mann–Whitney *U* test or the chi-square test as appropriate. All tests were two-sided, and a *P* value <0.05 was significant.

## Results

This section presents the baseline characteristics of the study population, the univariate predictive performance of each index, and the results of the multivariate logistic regression and machine learning models. Model performance was evaluated in the independent test cohort and compared across all algorithms.

### Study population characteristics

A total of 1,944 patients were enrolled from the two centers, including 1,437 in the training set and 507 in the test set. Gallstones were present in 222 patients (15.4%) in the training set and 88 patients (17.4%) in the test set (Table 2; Table S1). In the training set, NLR showed the highest predictive performance for gallstone occurrence, with an AUC of 0.627, followed by ABSI (0.615) and STR_area (0.610) (Table S2). The detailed distribution of CT indications was provided in Table S3. Among patients with gallstones detected on CT, 248 had isolated gallbladder stones, 28 had isolated bile duct stones, and 34 had concurrent gallbladder and bile duct stones (Table S4). The mean numbers of follow-up CT examinations were 4.37 and 7.46 in the gallstone and non-gallstone groups in Center 1, respectively, and 4.16 and 6.72 in the gallstone and non-gallstone groups in Center 2, respectively. Among patients who developed gallstones, the median interval from the initial CT examination to the first detection of gallstones was 1.12 years in Center 1 and 1.19 years in Center 2 (Table S5).

**Table 2.** Baseline characteristics of the training and test sets.

Variable	Training set (*n* = 1437)	Test set (*n* = 507)	*P* value
Age	63 (55–72)	63 (55–71)	0.664
Male	699 (48.6%)	266 (52.5%)	0.139
WC (cm)	91.8 (85.0–99.0)	94.3 (85.8–100.0)	0.007
Height (m)	1.65 (1.60–1.70)	1.67 (1.60–1.72)	0.109
Weight (kg)	65 (58–73)	68 (60–75)	0.000
NEUT	3.92 (2.86–5.58)	3.78 (2.865–5.285)	0.457
LYMPH	1.60 (1.18–2.09)	1.7 (1.29–2.21)	0.017
Blood glucose	5.78 (5.10–6.62)	5.72 (5.075–6.95)	0.612
TG	1.36 (1.00–1.92)	1.3 (0.97–1.82)	0.002
HDL_C	1.13 (0.93–1.36)	1.12 (0.94–1.38)	0.606
TC	4.67 (4.00–5.33)	4.72 (4.04–5.45)	0.034
TBIL	13.1 (9.8–17.4)	14.6 (11.4–19.05)	0.188
IBIL	9.4 (6.5–13.2)	11.2 (8.35–14.5)	0.016
Hypertension	494 (34.4%)	224 (44.2%)	0.000
Diabetes	286 (19.9%)	140 (27.6%)	0.000
Gallstones	222 (15.4%)	88 (17.4%)	0.313

WC waist circumference, NEUT neutrophil count, LYMPH lymphocyte count, TG triglycerides, HDL_C high-density lipoprotein cholesterol, TC total cholesterol, TBIL total bilirubin, IBIL indirect bilirubin.

### Multivariate logistic regression model

Based on the training set (222 in the gallstone group and 1,215 in the non-gallstone group), this study used a weighted forward stepwise logistic regression (Bayesian information criterion; weight for the positive group = number of negative cases/number of positive cases ≈ 5.47) to select predictive variables. The results of the fivefold cross-validation are presented in Table S6.

On the full training set, the weighted forward stepwise regression finally included six predictors: STR_area, NLR, Muscle_area, ABSI, TBIL, and Weight. Multivariate logistic regression showed that all variables were statistically significant (*p* < 0.01) (Table S7).

The final model was validated in the independent test set (*n* = 507). Using the optimal Youden threshold from the training set (0.492) for classification, the model achieved an AUC of 0.675, a sensitivity of 0.716, a specificity of 0.628, and an accuracy of 0.643.

### Machine learning models

Five machine learning models were constructed on the training set and compared with multivariate logistic regression (baseline model). Table 3 presents the predictive performance of each model on the training set and the independent test set. The ET model performed the best, with a test set AUC of 0.772 (95% confidence interval [CI]: 0.714–0.822). Random forest (RF) ranked second, with a test set AUC of 0.733 (95% CI: 0.675–0.786) (Figure 4). Repeated fivefold cross-validation with 50 different random seeds was performed on the training cohort. The ET model showed stable performance across different random states, with a mean AUC of 0.810 ± 0.015. The PR curves and confusion matrices of the five machine learning models are presented in Figure S1. The ET model achieved the highest average precision (AP = 0.642), followed by RF (AP = 0.615), CatBoost (AP = 0.570), SVM (AP = 0.565), and XGBoost (AP = 0.530). The confusion matrices demonstrated different classification patterns among the models, with ET showing a relatively balanced performance between positive and negative predictions.

**Figure 4.**
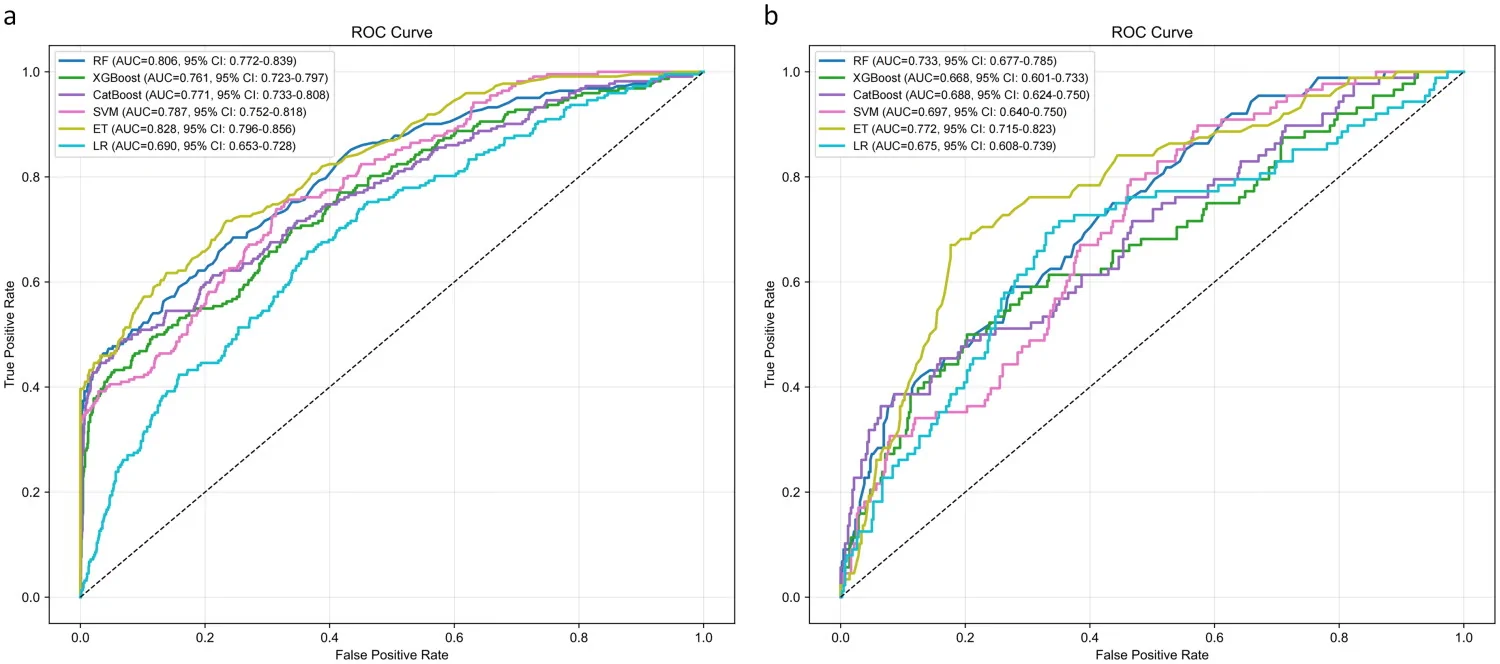
ROC curves of logistic regression and machine learning models for predicting gallstone occurrence. (a) Training set. (b) Test set. ROC receiver operating characteristic.

**Table 3.** Comparison of machine learning model results.

Model	Cohort	AUC (95% CI)	Accuracy	Sensitivity	Specificity
Logistic regression	Training	0.690 (0.652–0.728)	0.639	0.658	0.635
	Test	0.675 (0.609–0.741)	0.643	0.716	0.628
Extra Trees	Training	0.828 (0.796–0.856)	0.824	0.617	0.862
	Test	0.772 (0.714–0.822)	0.791	0.671	0.816
Random forest	Training	0.806 (0.772–0.839)	0.744	0.685	0.755
	Test	0.733 (0.675–0.786)	0.637	0.648	0.635
SVM	Training	0.787 (0.752–0.818)	0.693	0.730	0.686
	Test	0.697 (0.639–0.750)	0.765	0.341	0.854
CatBoost	Training	0.771 (0.733–0.808)	0.814	0.545	0.863
	Test	0.688 (0.622–0.752)	0.704	0.511	0.745
XGBoost	Training	0.761 (0.723–0.797)	0.811	0.500	0.867
	Test	0.668 (0.599–0.735)	0.793	0.307	0.895

AUC area under the receiver operating characteristic curve, CI confidence interval, SVM Support Vector Machine.

The SHAP analysis revealed significant differences in the contribution of each variable to the ET model’s predictions, with STR_area, age, and ITR_volume ranking among the top in importance (Figure 5). The correlation network plot showed that the features included in the model generally had low intercorrelations, with only a few moderate correlations. Specifically, ITR_volume was moderately positively correlated with ABSI and weight, while hypertension was moderately negatively correlated with weight. The correlations among the remaining variables were weak.

**Figure 5.**
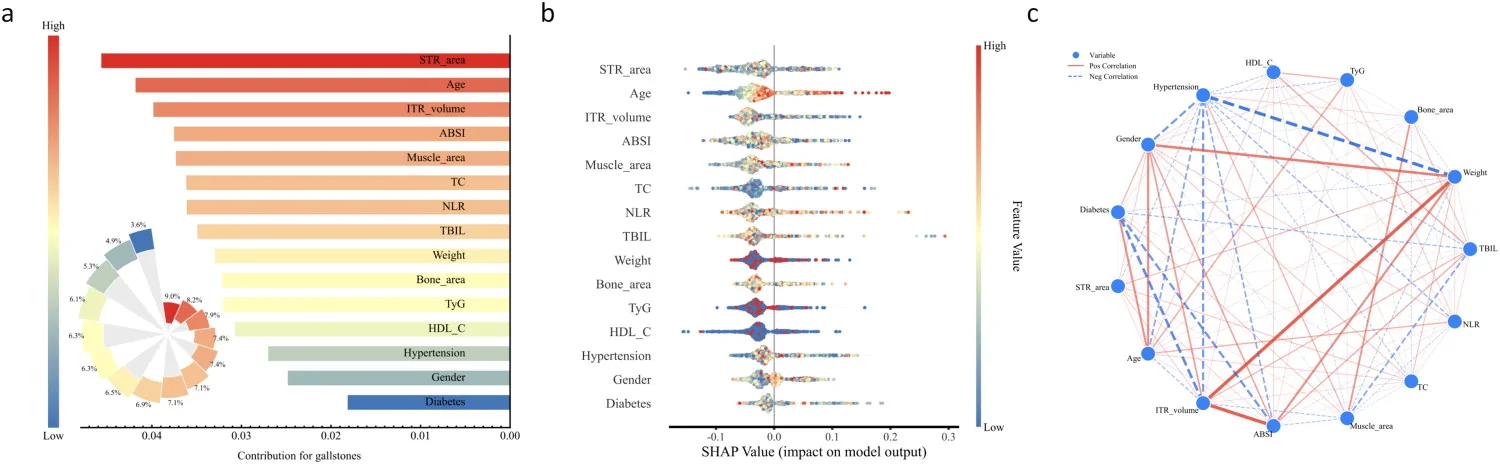
Feature importance and correlation analysis for gallstones prediction. (a) Contribution ranking of features based on Extra Trees (ET) prediction model. (b) SHAP beeswarm plot illustrating the distribution of the impact of each feature on model output, where each point represents a patient sample. (c) Correlation network diagram demonstrating the interrelationships among the predictive variables, with solid red lines representing positive correlations and dashed blue lines representing negative correlations (line thickness indicates correlation strength).

### Sensitivity analysis

In the sensitivity analysis restricted to patients with concordant CT and ultrasonography findings or surgical confirmation of gallstones, the ET model maintained comparable performance, achieving an AUC of 0.756 (95% CI: 0.655–0.840) in the sensitivity analysis cohort (Table S8). In another sensitivity analysis excluding patients with isolated bile duct stones, the ET model showed comparable performance, with an AUC of 0.767 (95% CI: 0.711–0.817) in the test set, compared with 0.772 (95% CI: 0.714–0.822) in the primary analysis (Table S9**).**

## Discussion

This study, based on a dual-center retrospective cohort, systematically compared the predictive performance of CT-based body composition indices, traditional anthropometric indices, and laboratory indices for the risk of gallstone occurrence, and further constructed a logistic regression model and five machine learning models. At the single-index level, the inflammatory marker NLR (AUC = 0.627), the anthropometric index ABSI (AUC = 0.615), and the body composition index STR_area (AUC = 0.610) performed best, representing the contributions of inflammation, obesity morphology, and fat distribution to gallstones, respectively. However, the performance of these three types of indices was limited, and their standalone application could hardly meet clinical decision-making needs. Among the logistic regression model and the machine learning models, the ET model achieved the best performance, with a test set AUC of 0.772, demonstrating the advantage of ensemble learning methods in risk prediction.

Univariate analysis showed that NLR had the highest predictive performance among all variables (AUC = 0.627). ABSI (AUC = 0.615) and STR_area (AUC = 0.610) ranked next, taking the first place among anthropometric indices and body composition indices, respectively. The AUCs of all three were below 0.63, indicating that a single index is difficult to use independently for clinical risk stratification. In multivariate analysis, each one-unit increase in NLR was associated with a 6.2% increase in gallstone risk. This suggests a driving role of inflammation in stone formation, which is consistent with previous reports of the association between gallstones and inflammatory markers [18]. Each 0.01 increase in ABSI was associated with a 59% increase in risk. Given a fixed height and weight, a high ABSI reflects a higher proportion of abdominal fat relative to peripheral tissue [30], and a ‘J-shaped’ relationship between ABSI and gallstones has been confirmed [31]. Each 0.1 increase in STR_area was associated with a 28% decrease in risk. Subcutaneous fat has traditionally been regarded as a storage depot for metabolically protective fat [32,33], and it may play a similar protective role in reducing the risk of gallstone occurrence. Although multivariate logistic regression improved the test set AUC to 0.675 by integrating multiple types of indices, its linear assumption makes it difficult to fully capture complex non-linear interactions and potential threshold effects among variables.

We further introduced five machine learning models. The results showed that the ET model performed best among all models, followed by RF (test set AUC = 0.733). SHAP analysis revealed that the three most important features contributing to machine learning models predictions were STR_area, age, and ITR_volume. Age, as a traditional risk factor, once again confirmed the increasing trend of gallstone occurrence with age. Intermuscular fat refers to fat deposits within skeletal muscle and is closely associated with insulin resistance and chronic low-grade inflammation [34,35]. Our finding that elevated ITR_volume is associated with an increased risk of gallstones suggests that ‘fat infiltration into muscle’ may indirectly promote stone formation by affecting systemic metabolic status.

Several previous studies have applied machine learning to gallstone prediction, providing useful context for our findings. Sarker et al. reported a higher AUC (0.828–0.877) [23], whereas Deng et al. (0.727) [24] and Zhao et al. (0.767) [25] reported AUCs comparable to or slightly lower than that of our ET model (0.772). However, it is important to note that all three cited studies were cross-sectional and focused on gallstone detection rather than longitudinal risk prediction. In contrast, our study is a dual-center retrospective cohort with a follow-up of at least 3 years, using the initial CT as the baseline and subsequently detected gallstones as the outcome. This longitudinal design establishes temporal relationships and enables risk stratification at baseline for future gallstone development, whereas cross-sectional studies can only capture associations at a single time point. Moreover, our model was externally validated in an independent center (*n* = 507), whereas the studies by Sarker et al. [23] and Zhao et al. [25] primarily relied on internal validation. These differences underscore the added value of the present work in terms of both study design and clinical applicability.

In this study, we found that among patients with available ultrasonography data, 77 cases (7.2%) were negative by CT but positive by ultrasound. These patients were classified into the non-gallstone group based on CT results in the primary analysis, suggesting that relying solely on CT for outcome determination may miss some gallstone cases and thus lead to potential outcome misclassification. The main reason for this misclassification is that ultrasound is more sensitive than CT for detecting small or sludge-like stones. In the primary analysis, we did not incorporate ultrasound into the outcome definition, primarily because this was a retrospective cohort and ultrasound was not a pre-specified examination in the original study protocol. Only 1,069 patients (approximately 53.6%) had ultrasound data during the observation period, and the timing of examinations mostly did not meet the same follow-up requirements as CT. If the outcome definition were modified based only on ultrasound results from a subset of patients while the remaining patients were still classified by CT, this would create inconsistent outcome ascertainment and could introduce bias related to the opportunity for or indication for ultrasound. To address this, we performed a sensitivity analysis including only patients with concordant CT and ultrasound findings. The AUC was 0.772 (95% CI: 0.714–0.822) in the primary analysis and 0.756 (95% CI: 0.655–0.840) in the sensitivity analysis. The two values were close, suggesting that the impact of this misclassification on the overall predictive performance of the model was limited and that the main conclusions remain robust. In addition, to assess whether the inclusion of bile duct stones influenced the primary results, we conducted a sensitivity analysis by excluding patients with isolated bile duct stones and redefining the remaining stone patients as the positive outcome. In this refined cohort, the ET model showed comparable performance to the primary analysis (AUC = 0.767, 95% CI: 0.711–0.817), confirming that the main results were not driven by this smaller subgroup. Collectively, these sensitivity analyses support the reliability and generalizability of the ET model’s predictive performance.

We also examined the difference in the number of follow-up CT examinations between the gallstone and non-gallstone groups. In the non-gallstone group, patients underwent at least one CT per year over the 3-year follow-up, whereas imaging follow-up ended for the gallstone group once stones were detected. Given that the median time from the initial CT to the first detection of gallstones was approximately 1.1 years, the lower number of follow-up CT examinations in the gallstone group was a direct consequence of the earlier endpoint event. This difference primarily reflected the inherent temporal structure of the cohort design, not differential surveillance intensity.

The clinical value of this study is mainly reflected in the following aspects. First, for patients who have already undergone abdominal CT, automated tools such as BOA can extract body composition parameters including STR_area and ITR_volume without additional scanning. Combined with routine laboratory indices, this enables refined risk stratification without increasing radiation exposure or medical costs. Second, in community screening settings where only basic anthropometric data are available, ABSI still provides moderate predictive performance and can serve as a simple preliminary screening tool. Third, the ET model demonstrates the advantage of non‑linear ensemble methods in improving prediction accuracy. In the future, it could be packaged as a clinical decision-support tool to help physicians identify high-risk individuals and develop personalized follow-up strategies.

This study has the following limitations. First, the retrospective design may have introduced selection bias. The requirement for repeated abdominal CT examinations in the inclusion criteria may have selected a population with higher comorbidity burdens or specific clinical indications. In our study cohort, the most common indications for the initial CT examination were abdominal pain or discomfort and tumor surveillance/follow-up, reflecting a clinical population rather than the general population. This factor should be considered when generalizing our findings to asymptomatic or community-based populations. Second, the diagnosis of gallstones in this study relied on abdominal CT, which may miss isodense or small stones, potentially leading to a small number of stone patients being misclassified into the non-stone group. However, since this was a retrospective study, ultrasonography data were not uniformly available, were non-standardized, and were performed at inconsistent time points; therefore, other examinations such as ultrasonography could not be used to supplement or verify the CT diagnosis. Although we performed sensitivity analyses and the results were similar to the primary analysis, these analyses still could not fully eliminate the impact of misclassification. In addition, because the timing of follow-up imaging was irregular, time-to-event analysis could not be reliably performed. Finally, lifestyle factors such as diet, physical activity, smoking, and alcohol consumption were not collected, which may have resulted in residual confounding.

## Conclusions

Based on a dual-center retrospective cohort, this study systematically compared the predictive performance of CT-based body composition indices, anthropometric indices, and laboratory indices for the risk of gallstone occurrence. In univariate analysis, the inflammatory marker NLR showed the highest predictive performance, followed by the anthropometric index ABSI and the body composition index STR_area. However, the predictive ability of each individual index was limited. Logistic regression and machine learning integrating multiple categories of indices effectively improved predictive performance, with the ET model performing best. The ET model, combined with automated body composition analysis, holds promise for assisting clinical risk stratification.

## Supplementary Material

Supplementary Material the clean version R2.docx

## Data Availability

The datasets used and/or analysed during the current study are available from the corresponding author on reasonable request
