# Supplementary material for: Predicting gallstones risk with body composition analysis and machine learning: a dual-center cohort study
Source: Ann Med. 2026 Oct 7;58(1):2742549. doi: 10.1080/07853890.2026.2742549 (PMC13647361; doi:10.1080/07853890.2026.2742549)
Supplement: Supplementary file 1 [file IANN_A_2742549_SM9396.docx]

**Appendix 1**

Center 1: The Siemens device utilized a tube current of 312 mA, tube voltage of 120 kVp, matrix size of 512×512, and a field of view (FOV) of 50 cm. The Toshiba device utilized a tube current of 300 mA, tube voltage of 120 kVp, matrix size of 512×512, and a FOV of 50 cm.

Center 2: The Siemens device utilized a tube current of 260 mA, tube voltage of 120 kVp, matrix size of 512×512, and a FOV of 50 cm.

**Appendix 2**

WC was measured per the International Diabetes Federation anatomical definition and approximated using bony landmarks on CT images. TotalSegmentator automatically identified the longitudinal positions of the inferior border of the lowest visible rib and the superior borders of the bilateral iliac crests. And the mid‑plane between them was taken as the standardized analysis slice. Subsequently, a self‑programmed Python algorithm extracted the body surface contour from the BOA segmentation results at this slice, and its closed perimeter was calculated as the imaging‑based WC.

**Appendix 3**

Hypertension: SBP ≥130 mmHg and/or DBP ≥85 mmHg, or a confirmed diagnosis of hypertension receiving treatment

Diabetes: fasting blood glucose ≥6.1 mmol/L, or 2‑hour post‑load blood glucose ≥7.8 mmol/L, or a confirmed diagnosis of diabetes receiving treatment

$$RFM:Males=64-[20*(\frac{Height\left( m \right)}{WC\left( m \right)})],Females=76-[20*(\frac{Height(m)}{WC(m)})]$$

$$BRI=364.2-365.5*\sqrt{1-{(\frac{WC(cm)}{2\pi})}^{2}/{(\frac{Height(cm)}{2})}^{2}}$$

$$BMI=\frac{Weight(kg)}{{Height(m)}^{2}}$$

$$ABSI=\frac{WC(m)}{{BMI}^{2/3}*{Height(m)}^{1/2}}$$

$$TyG = ln (TG (mmol/L)\times Glu (mmol/L)\times797.13）$$

$$TyG-ABSI=TyG*ABSI$$

$$NLR=\frac{\mathrm{NEUT}}{\mathrm{LYMPH}}$$

**Appendix 4**

Logistic regression (LR) is a conventional statistical classification method that estimates the probability of an outcome based on a linear combination of predictor variables through the logistic function. In this study, LR was included as a baseline model for comparison with machine learning algorithms. ET is an ensemble learning algorithm based on randomized decision trees, which reduces model variance by introducing additional randomness in feature selection and node splitting. RF constructs multiple decision trees through bootstrap sampling and random feature selection, and combines their predictions for final classification. SVM performs classification by identifying the optimal decision boundary that maximizes the margin between different classes and can handle nonlinear relationships through kernel functions. XGBoost is a gradient boosting tree algorithm that sequentially builds decision trees to reduce prediction errors, thereby capturing complex nonlinear relationships and interactions among variables. CatBoost is also a gradient boosting tree algorithm and improves model stability and generalizability through techniques such as ordered boosting.

These algorithms were selected because they represent different modeling strategies and allow evaluation of the relationships between study variables and outcomes from different perspectives. All algorithms were compared under the same data preprocessing, cross-validation, and hyperparameter optimization framework, and the algorithm with the best predictive performance was selected as the final model.

**Appendix 5**

Image segmentation was performed using the Body and Organ Analysis (BOA) tool deployed through a Docker container (Docker version: 28.4.0) under the Ubuntu 24.04.3 LTS environment.

Image post-processing and extraction of body composition parameters from BOA outputs were performed using Python (version: 3.7.12). The main libraries included NiBabel (version: 4.0.2), SimpleITK (version: 2.1.1.1), NumPy (version: 1.21.5), Pandas (version: 1.3.5), OpenCV (version: 4.12.0), PyTorch (version: 1.13.1), and tqdm (version: 4.67.1).

Machine learning models were developed using the Scikit-learn Pipeline framework with Python (version: 3.10.9) and scikit-learn (version: 1.0.2). Additional machine learning libraries, including XGBoost (version: 1.6.2) and CatBoost (version: 1.2.7), were used for model construction. A fixed random seed (random seed = 888) was applied during model training to ensure reproducibility.

All computational procedures were performed on a workstation equipped with an AMD Ryzen 7 9700X CPU (8 cores/16 threads), 48 GB RAM, and an NVIDIA GeForce RTX 5080 GPU (16 GB memory).

**TableS1** Normality assessment and descriptive statistics of continuous variables in the training and test sets

| Variable | Training set (n = 1437) | | Test set (n = 507) | |
| --- | --- | --- | --- | --- |
|  | Mean ± SD | Min–Max | Mean ± SD | Min–Max |
| Age | 63±13 | 21-94 | 63±12 | 19-92 |
| WC (cm) | 92.04±10.71* | 60.78-138.21 | 93.57±11.27 | 62.75-134.27 |
| Height (m) | 1.66±0.076 | 1.4-1.9 | 1.66±0.084 | 1.35-1.88 |
| Weight (kg) | 65.96±12.00 | 38-140 | 68.38±13.47 | 34-130 |
| NEUT | 4.80±3.46 | 0.39-53.72 | 4.67±3.07 | 0.23-28.51 |
| LYMPH | 1.68±0.75 | 0.10-10.10 | 1.78±0.70 | 0.23-5.79 |
| Blood glucose | 6.43±2.57 | 1.97-28.97 | 6.50±2.53 | 3.20-30.59 |
| TG | 1.66±1.44 | 0.25-24.74 | 1.94±2.24 | 0.27-20.51 |
| HDL_C | 1.17±0.37 | 0.11-3.12 | 1.18±0.37 | 0.23-3.42 |
| TC | 4.70±1.14 | 0.44-16.26 | 4.82±1.33 | 0.98-18.72 |
| TBIL | 17.16±27.38 | 2.0-522.4 | 19.00±22.52 | 5.10-266.00 |
| IBIL | 11.43±13.22 | 0.0-259.0 | 13.16±10.65 | 0.0-118.70 |

WC waist circumference, NEUT neutrophil count, LYMPH lymphocyte count, TG triglycerides, HDL_C high-density lipoprotein cholesterol, TC total cholesterol, TBIL total bilirubin, IBIL indirect bilirubin. Normality was assessed using the Kolmogorov-Smirnov test; * indicates a normal distribution, and variables without an asterisk were non-normally distributed.

**Table S2** Univariate performance evaluation

| Variable | Gallstone | Non-gallstone | *P* value | AUC (95%CI) |
| --- | --- | --- | --- | --- |
| NLR | 3.250 (1.914-5.841) | 2.265 (1.567-3.594) | 0.000 | 0.627 (0.586-0.668) |
| ABSI | 0.088 (0.084-0.092) | 0.086 (0.082-0.09) | 0.000 | 0.615 (0.576-0.654) |
| STR_area | 0.39 (0.3-0.475) | 0.442 (0.339-0.541) | 0.000 | 0.610 (0.571-0.648) |
| Age | 67 (58-77) | 63 (55-71) | 0.000 | 0.605 (0.564-0.646) |
| TC | 4.58 (3.750-5.148) | 4.700 (4.060-5.350) | 0.008 | 0.564 (0.522-0.606) |
| Bone_area (cm^2^) | 28.587 (24.902-33.405) | 27.334 (24.482-31.392) | 0.007 | 0.563 (0.52-0.605) |
| ITR_volume | 0.073 (0.053-0.095) | 0.065 (0.051-0.082) | 0.000 | 0.563 (0.518-0.607) |
| Gender | 125 (56.3%) | 574 (47.2%) | 0.013 | 0.545 (0.51-0.581) |
| Hypertension | 93 (41.9%) | 401 (33.0%) | 0.011 | 0.544 (0.509-0.58) |
| HDL_C | 1.105 (0.900-1.34) | 1.140 (0.930-1.370) | 0.007 | 0.538 (0.497-0.58) |
| Muscle_area (cm^2^) | 102.034 (81.066-124.882) | 104.837 (88.378-127.079) | 0.128 | 0.531 (0.489-0.574) |
| TyG | 8.773 (8.398-9.186) | 8.837 (8.464-9.215) | 0.357 | 0.53 (0.489-0.571) |
| Diabetes | 55 (24.8%) | 231 (19.0%) | 0.049 | 0.529 (0.498-0.559) |
| TBIL | 13.1 (9.9-17.2) | 13.4 (9.5-18.2) | 0.003 | 0.517 (0.474-0.561) |
| Weight | 65 (58-73) | 65 (60-72.75) | 0.751 | 0.501 (0.458-0.543) |

NLR neutrophil-to-lymphocyte ratio, ABSI A Body Shape Index, STR_area SAT/TAT area ratio, TC total cholesterol, ITR_volume IMAT/TAT volume ratio, HDL_C high-density lipoprotein cholesterol, TyG triglyceride-glucose index, TBIL total bilirubin, SAT subcutaneous adipose tissue, IMAT intermuscular adipose tissue, TAT total adipose tissue.

**Table S3** Clinical indications for the initial abdominal CT examinations in the two centers

| **Clinical indication for initial abdominal CT** | **Center 1, n (%)** | **Center 2, n (%)** |
| --- | --- | --- |
| Abdominal pain or abdominal discomfort | 713 (49.62%) | 161 (31.76%) |
| Tumor surveillance or follow-up | 381 (26.51%) | 115 (22.68%) |
| Other indications* | 343 (23.87%) | 231 (45.56%) |
| Total | 1,437 (100%) | 507 (100%) |

Note:*Other indications included various reasons, such as routine health examinations and follow-up for other diseases.

**Table S4** Anatomical distribution of CT-detected gallstones in the two centers

| **Stone location** | **Center 1, n (%)** | **Center 2, n (%)** |
| --- | --- | --- |
| Isolated gallbladder stones | 177 (79.73%) | 71 (80.68%) |
| Isolated bile duct stones | 19 (8.56%) | 9 (10.23%) |
| Concurrent gallbladder and bile duct stones | 26 (11.71%) | 8 (9.09%) |

**Table S5** Follow-up abdominal CT examinations and time to first detection of gallstones in the two centers

| **Variable** | **Center 1** | | **Center 2** | |
| --- | --- | --- | --- | --- |
|  | Gallstone group | Non-gallstone group | Gallstone group | Non-gallstone group |
| Mean number of follow-up CT examinations per patient | 4.37 | 7.46 | 4.16 | 6.72 |
| Median interval from initial CT to first detection of gallstones (years) | 1.12 | NA | 1.19 | NA |

Note: The median interval from the initial CT examination to the first detection of gallstones was calculated only among patients in the gallstone group.

**Table S6** 5-fold cross-validation results of the weighted logistic regression model

| Model | AUC | Sensitivity | Specificity | Accuracy | F1 score |
| --- | --- | --- | --- | --- | --- |
| Weighted | 0.667±0.039 | 0.622±0.183 | 0.682±0.186 | 0.674±0.127 | 0.376±0.048 |

**Table S7** Variables included in the multivariate logistic regression model

| Variable | β | OR(95%CI)* | *P* value |
| --- | --- | --- | --- |
| STR_area | -3.234 | 0.724 (0.670–0.782) | 0.000 |
| NLR | 0.060 | 1.062(1.042-1.081) | 0.000 |
| Muscle_area | -0.015 | 0.985(0.981-0.989) | 0.000 |
| ABSI | 46.292 | 1.59 (1.38–1.82) | 0.000 |
| TBIL | 0.007 | 1.007(1.004-1.011) | 0.000 |
| Weight | 0.018 | 1.018(1.008-1.029) | 0.000 |

Note:*The OR for STR_area corresponds to each 0.1 increase; the OR for ABSI corresponds to each 0.01 increase; the ORs for the remaining variables correspond to each 1-unit increase. OR Odds Ratio, CI confidence interval, STR_area SAT/TAT area ratio, NLR neutrophil-to-lymphocyte ratio, ABSI A Body Shape Index, TBIL total bilirubin, SAT subcutaneous adipose tissue, TAT total adipose tissue.

**Table S8** Agreement between CT-based diagnosis and ultrasonography or surgical findings

|  | **Center 1** | | **Center 2** | |
| --- | --- | --- | --- | --- |
| **Reference standard** | **CT-positive** | **CT-negative** | **CT-positive** | **CT-negative** |
| Surgery-positive* | 5 | 0 | 2 | 0 |
| Ultrasound-positive | 45 | 56 | 34 | 21 |
| Ultrasound-negative | 40 | 563 | 25 | 285 |

*Surgical records were available only among patients with gallstones; therefore, no surgically confirmed negative cases were identified. All patients with surgical records underwent abdominal ultrasonography during the observation period.

**Table S9** Sensitivity analysis excluding patients with isolated bile duct stones

| **Dataset** | **AUC (95% CI)** | **Accuracy** | **Sensitivity** | **Specificity** |
| --- | --- | --- | --- | --- |
| Training set (excluding isolated bile duct stones) | 0.811 (0.774–0.843) | 0.842 | 0.567 | 0.887 |
| Original training set | 0.828 (0.796–0.856) | 0.824 | 0.617 | 0.862 |
| Test set (excluding isolated bile duct stones) | 0.767 (0.711–0.817) | 0.797 | 0.506 | 0.851 |
| Original test set | 0.772 (0.714–0.822) | 0.791 | 0.671 | 0.816 |

**Figure S1.** Precision-recall curves and confusion matrix of the five machine learning models. (a) Support Vector Machine (SVM); (b) Random Forest (RF); (c) Extra Trees (ET); (d) CatBoost; and (e) XGBoost. (f) Confusion matrices of the five machine learning models. GS gallstones, AP average precision.
